# The Quality of Coronary Artery Bypass Grafting Videos on YouTube

**DOI:** 10.7759/cureus.44281

**Published:** 2023-08-28

**Authors:** Bradley M Nus, Kylie Wu, Trey Sledge, Grant Torres, Sai Kamma, Sanjana Janumpally, Syed Gilani, Scott Lick

**Affiliations:** 1 Cardiology, University of Texas Medical Branch at Galveston, Galveston, USA; 2 Cardiology, Texas College of Osteopathic Medicine, Fort Worth, USA; 3 Cardiology, Kansas City University, Kansas City, USA; 4 Cardiothoracic Surgery, University of Texas Medical Branch at Galveston, Galveston, USA

**Keywords:** quality of healthcare videos, youtube videos, coronary artery bypass grafting (cabg), cardiology, accreditation, patient education

## Abstract

Objective

YouTube (YouTube LLC, San Bruno, California, United States), one of the most accessed sites on the internet, has become a widespread source of healthcare information for patients. Videos about coronary artery bypass grafts (CABG) have accrued tens of millions of views on the platform, yet their educational quality is unknown. This study investigates the educational landscape of videos regarding CABG procedures on YouTube.

Methods

YouTube was queried for “Coronary Artery Bypass Graft Surgery” and “Coronary Artery Bypass Graft Procedure”. After applying exclusion criteria, 73 videos were assessed. Two independent reviewers rated the material with the Global Quality Scale (GQS) (5 = high quality, 0 = low quality) to judge educational value. A ratio of view count to days since upload was applied to assess video popularity. Source, modality, and date of upload were recorded for each video as well.

Results

An average GQS score of 2.94 was found, indicating poor educational quality of the 73 YouTube videos on CABG procedures. Videos uploaded by physicians (56/73; 76.7%) had a significantly higher average GQS score than those uploaded by non-physicians (p<0.001). When content was grouped by delivery method, physician-led presentations (24/73 or 32.9%) produced the highest average GQS score of 3.35; conversely, patient-friendly delivery methods (18/73 or 24.7%) yielded the lowest average GQS score of 2.36 (p<0.001). Neither the view ratio nor the days since upload significantly correlated with the educational quality of the video.

Conclusion

Although CABG videos are readily available on YouTube, they often contain considerable biases and misleading information. With online sources for healthcare education now commonplace, physicians must be aware of the vast quantities of low-quality videos patients often encounter when weighing different treatment options. Further analysis of CABG videos on YouTube may allow physicians to ameliorate this gap by producing videos that are not only high quality but highly viewed on the platform.

## Introduction

Internet use has increased in prevalence around the world as countries continue to expand their online infrastructure [1]. Recently, coronavirus disease 2019 (COVID-19) accelerated this process by forcing companies to transition their workforce to virtual environments [2]. This trend is notable in healthcare too, as up to 80% of internet users search for healthcare content online [3,4]. Rather than browse long convoluted articles, many individuals watch videos for concise lessons on health-related topics.

With over two billion monthly viewers, YouTube (YouTube LLC, San Bruno, California, United States) is the largest online video platform, and a popular source for medical information [5,6]. However, this extensive catalog of healthcare content is not subject to any peer review process and is often uploaded by creators who lack credibility on the topics they are presenting [7,8]. Furthermore, YouTube employs a personalized algorithm that guides users toward content they agree with, regardless of its validity [9]. This facilitates online echo chambers rife with healthcare misinformation that undermines the public’s confidence in scientific communities [10]. An example of this was the proliferation of inaccurate healthcare information related to anti-vaccination sentiments and alternative treatments for COVID-19 across different social media platforms in reaction to the pandemic [11,12].

Despite this disparity between content quality and quantity, many patients highly value the healthcare information they find online. A previous survey found that the Internet was the second most popular source of healthcare information with 11% of individuals deferring to online information instead of consulting their doctor [13]. Clinicians must be aware of this, as medical misinformation can strain the patient-physician relationship, and influence patients’ decisions regarding different treatment options [14,15]. These concerns have prompted many investigators to assess the quality of healthcare content on YouTube, often revealing its misleading nature [7,16].

Coronary artery bypass grafting (CABG) continues to be the most ubiquitous cardiac surgery in the world [17]. Approximately 340,000 CABG procedures are performed each year in the United States alone [18]. This life-changing surgery has accrued tens of millions of views on YouTube as many patients rely on videos to educate themselves on the topic. Despite the increasing popularity of CABG videos, to our knowledge, no previously published studies have assessed their educational quality. Therefore, in response to the recent concerns regarding YouTube healthcare content, the purpose of this study was to define the educational landscape of CABG videos on the platform.

## Materials and methods

Search strategy

**Figure 1:**
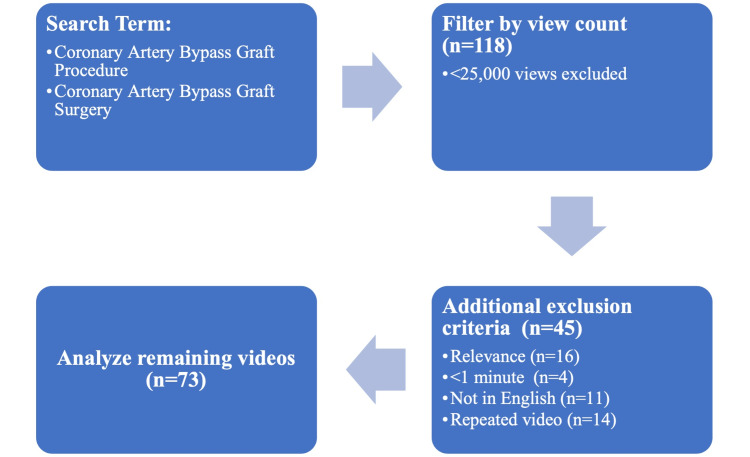
Methodology for CABG YouTube* video selection flowchart *YouTube LLC, San Bruno, California, United States CABG: coronary artery bypass graft

Our methodology was based on previous research studies that conducted similar analyses of health-related YouTube content [7,19,20]. A new YouTube (www.youtube.com) account was created, and Google Chrome (Google LLC, Mountain View, California, United States) was set to "incognito mode" to eliminate any personalized tailoring of the YouTube algorithm to the relevant search. Two distinct phrases were entered into YouTube’s search engine to identify relevant videos on May 18, 2020: “Coronary Artery Bypass Graft Surgery” and “Coronary Artery Bypass Graft Procedure”. The filter option on YouTube was then set to “filter by view count” which organizes the videos in descending order from highest to lowest view count at the time of the search. Videos with less than 25,000 total views were excluded as these were not popular enough to warrant analysis. This left a total of 118 videos for evaluation, before applying additional exclusionary criteria. Videos that were not in English (n=11), not relevant to CABG procedures (n=16), less than one minute in duration (n=4), and duplicates (n=14) were also excluded based on previous studies [21]. Only English videos were included as English is the universal language adopted by many countries around the world [22]. Videos less than one minute were excluded as they generally did not contain enough information to properly analyze the content. After applying all exclusion criteria, 73 videos were left for final scoring and analysis (Figure 1).

Video assessment

The selected videos were scored in a similar process implemented by previous studies [7,20]. All 73 videos were rated by two independent reviewers (BN, KW) in such a way that every video was scored twice. This was done to minimize any personal bias regarding video quality, as well as to increase reproducibility of the scoring process. Both reviewers were medical students who were well versed in the CABG procedure. The Global Quality Scale (GQS) score, a modified DISCERN score, was used to judge video quality as no unique video rating scale has been standardized at this time[23,24]. All videos were assessed with the GQS score by quantifying which of the five key criteria were met (Table 1). Every criterion met awarded 1 point to the video in question, with the sum of this score estimating the overall quality of the video. Videos that contained high levels of biased, misleading, and poorly presented information from a non-credible source received a GQS score of 0. Videos with a GQS score of 5 were considered to have excellent quality and contain unbiased information from a credible source (Table 2). Generally, a GQS score of 0-3 was considered to have an overall poor/moderate quality, while a GQS score of 4-5 was considered to have an overall high/excellent quality.

**Table 1:** Global Quality Scale criteria used to score CABG videos on YouTube* CABG: coronary artery bypass graft *YouTube LLC, San Bruno, California, United States

Quality of information (1 point for Yes, 0 points for No)
1. Are the aims clear and achieved?
2. Are reliable sources of information used?
3. Is the information balanced and unbiased?
4. Are additional sources of information provided for patient reference?
5. Are areas of uncertainty mentioned?

**Table 2:** Assessment of CABG video quality based on total GQS score CABG: coronary artery bypass graft; GQS: Global Quality Scale *YouTube LLC, San Bruno, California, United States

Total GQS score	Description
1	Poor quality, misleading, clear bias
2	Subpar quality, may be misleading, some bias
3	Moderate quality, may contain bias, not all information accurate
4	Good quality, generally unbiased, missing some information and references
5	Excellent quality, accurate, unbiased

Descriptive statistics including video title, hyperlink, duration, total likes and dislikes, total comments, days since upload, view count, content, source, and delivery method were recorded. Videos were then organized into three categories based on the delivery method of the video. These categories included videos that were patient-friendly modalities, live procedures, or presentations. The rationale behind these categorizations was to better characterize the current catalog of content regarding CABG procedures on YouTube as well as determine if variations in the content’s educational quality existed because of the chosen modality. Patient-friendly modalities included animations, news reports, and patient accounts, as this information was often straightforward and easiest to interpret without any prior medical knowledge. Live procedures were considered the least consumer-friendly modality as they often required a medical background to understand the video content. This is because videos of live procedures often contained little to no description of the content being presented and required a complex understanding of the surgery being performed and knowledge of key anatomical landmarks to comprehend the video. Presentations fell in the middle of comprehensibility with some including advanced analyses of CABG procedures, and others containing foundational knowledge for a broad audience. Content was also divided into two groups based on whether a physician was present in the video to substantiate any claims made about the procedure.

The like-to-dislike ratio (total number of likes/total number of dislikes) was calculated to judge community approval as seen in previous studies [20]. The view ratio (total number of views/days since upload) was also calculated to assess the video's popularity while correcting for the amount of time the video had been on the platform [19,25].

Statistical analysis

Statistical analysis was performed with IBM SPSS Statistics for Windows, Version 25.0 (2017; IBM Corp., Armonk, New York, United States). The normality of the data was tested with the Shapiro-Wilks test. Continuous variables were reported as mean, median, and standard deviation, while categorical variables were reported as frequencies and percentages. All numerical results were rounded to two decimal places. An intraclass correlation coefficient (ICC) was calculated between reviewers to evaluate the agreement of the independent scores. The interpretation of the ICC was based on previously established criteria, which suggested a value above 0.8 was considered “excellent”, between 0.6 and 0.8 was considered “substantial”, between 0.4 and 0.6 was considered “moderate”, and below 0.4 was considered “poor” [26]. Pairwise comparisons between average GQS scores and video characteristics were evaluated using the Mann-Whitney U test. A p-value <0.05 was considered statistically significant. A single-factor analysis of variance (ANOVA) test was used for comparisons among three or more variables, followed by post-hoc tests with the Bonferroni method. The Pearson correlation coefficient was used to analyze relationships between GQS score and video characteristics.

Ethics statement

All information was taken from existing data that was publicly available and is therefore exempt from institutional review board approval. 

## Results

A total of 73 videos that contained medical information pertinent to patients considering the procedure were included for final analysis after excluding 45 based on preset criteria to ensure adequate relevance. The average GQS score of all 73 videos analyzed was 2.94, indicating an overall poor educational quality (standard deviation (SD): 0.8). The GQS scores awarded to each video ranged from 0 to 4 with no video obtaining a perfect score of 5 from either independent reviewer.

Table 3 summarizes the descriptive data of the videos evaluated. Videos, on average, were 2,638.8 days or 7.2 years old (SD: 1,209.2 days) with a range of 384 to 5,025 days old. The total amount of views for all analyzed videos was 45,665,709, with an average of 625,558 views per video (SD: 2,246,334.7), and a range of 26,454-18,306,115 views. Additionally, the median view count per video was 107,055. The average time per video was 544.3 seconds or about nine minutes (SD: 798.3 seconds) and ranged from 60 to 6,166 seconds. The total amount of likes of all included videos was 170,354, with an average of 2,334 likes per video (SD: 7,748.7), ranging from 0 to 59,000 likes. The total amount of dislikes of all included videos was 16,619, with an average of 228 dislikes per video (SD: 806.4), and a range of 0-6,400 dislikes. The total amount of comments for included videos was 8,450, with an average of 115.8 comments per video (SD: 378.0), ranging from 0 to 2,850 comments.

**Table 3:** Descriptive characteristics of YouTube videos on CABG *YouTube LLC, San Bruno, California, United States CABG: coronary artery bypass graft; GQS: Global Quality Scale

	Mean ( ±SD) (n=73)	Range (min – max)
Days since upload	2,638.8 ± 1,209.2	384 – 5,025
View count	625,558 ± 2,246,334.7	26,454 – 18,306,115
Duration (sec)	544.3 ± 798.3	60 – 6,166
Likes	2,334 ± 7,748.7	0 – 59,000
Dislikes	228 ± 806.4	0 – 6,400
Comments	115.8 ± 378.0	0 – 2,850
Like-to-dislike ratio	16.9 ± 15.7	3.8 – 77.8
View ratio	247.8 ± 761.3	7.2 – 6,041
GQS Score	2.94 ± 0.8	0 – 4

**Figure 2:**
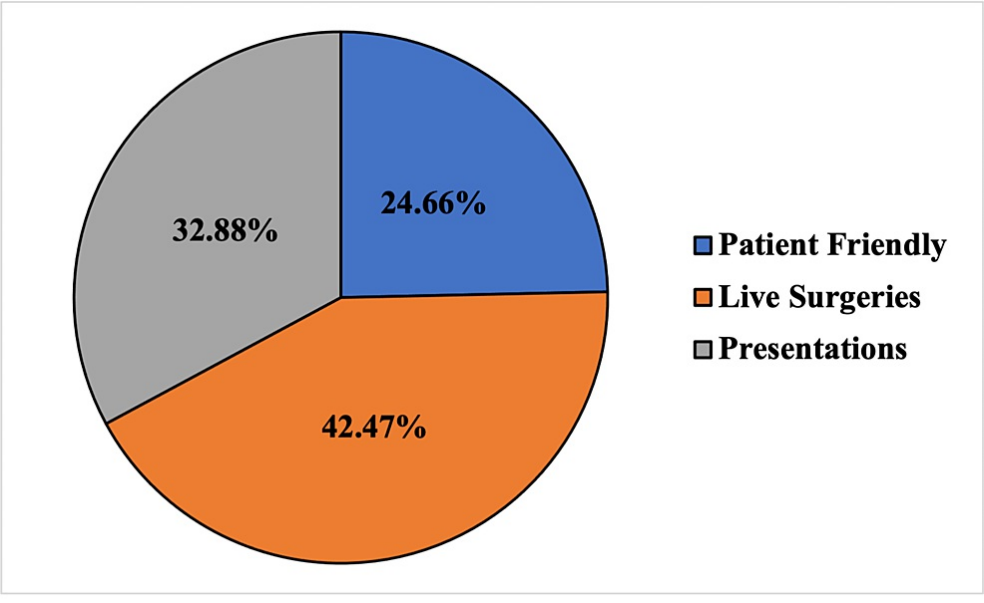
Video content stratified by delivery method

Patient-friendly modalities (news reports, animations, patient stories) accounted for 24.7% (18/73) of videos, live surgeries accounted for 42.5% (31/73) of videos, and 32.9% (24/73) of videos were presentations (Figure 2).

**Figure 3:**
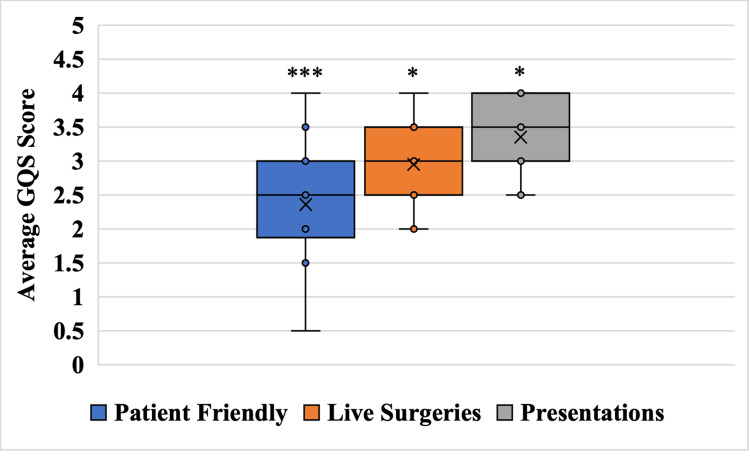
Average GQS score vis-a-vis video delivery method * p-value < 0.05, *** p-value <0.001 GQS: Global Quality Scale

When video quality was compared to delivery method, presentations produced the highest average GQS score of 3.35 (SD: 0.52); conversely, patient-friendly delivery methods yielded the lowest average GQS score of 2.36 (SD: 0.82) (p<0.0001). Live surgeries had an intermediate average GQS score of 2.95 (SD: 0.49) which was significantly higher than patient friendly videos (p<0.01) and significantly lower than presentations (p<0.01) (Figure 3).

**Figure 4:**
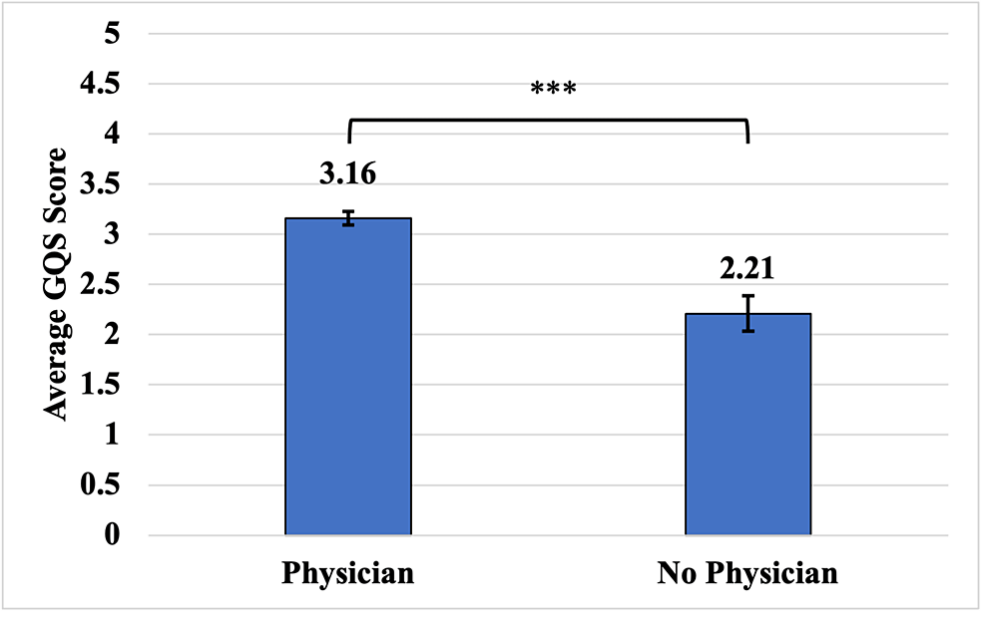
Average GQS score vis-a-vis presence or absence of a physician in the video *** p-value <0.001 GQS: Global Quality Scale

Among the 73 videos analyzed, 76.7% (56/73) included a physician substantiating the claims made in the video, while the remaining 23.3% (17/73) did not. Content with at least one physician present in the video had an average GQS score of 3.16 (SD: 0.26). This was significantly higher than videos that did not contain a physician, which had an average GQS score of 2.21 (SD 0.53) (p<0.0001) (Figure 4).

The average view ratio of the 73 videos analyzed was 247.8 views per day (SD: 761.3), ranging from 7.2 to 6,041 views per day. There was no significant difference in the average GQS score between videos with view ratios greater than 50 views per day (37/73) and videos with view ratios less than 50 views per day (36/73) (p=0.57).

After excluding videos with 0 likes and dislikes (n=4), the average like-to-dislike ratio was 16.9 likes per dislike (SD: 15.7), with a range of 3.8-77.8 likes per dislike. No significant difference in the average GQS score was found between the videos with like-to-dislike ratios above 10 likes per dislike (43/69) and videos with like-to-dislike ratios below 10 likes per dislike (26/69) (p=0.54). No significant correlation was found between the days since upload and the average GQS score of the videos. Similarly, video duration and average GQS scores were not significantly correlated. The ICC for GQS scores was 0.52, which indicated moderate agreeability between the independent reviewers.

## Discussion

YouTube is currently the most popular video platform on the internet, and patients are increasingly relying on it as a source of healthcare information [4,13,27]. Recent studies have revealed that 45% of patients research treatment options online prior to consulting their physician [28]. This trend of increased dependence on the internet for patient education, coupled with the lack of peer-reviewed videos on YouTube, facilitates the spread of misinformation and strains the physician-patient relationship [10,11,14]. However, clinicians who understand the educational landscape of YouTube medical content can direct patients toward reliable sources of information and combat common misconceptions that are ubiquitous on the platform. Furthermore, motivated physicians may upload their own educational content to better enable those seeking additional information outside of the often-brief doctor’s visit. This study revealed that the majority of YouTube videos regarding CABG had low educational quality and contained high levels of bias and misinformation. These findings are consistent with previous studies regarding other medical procedures on YouTube [16,29].

Although an average GQS score of 2.94 exposes the low reliability of YouTube CABG content overall, different results are shown when video modalities are analyzed separately. Patient-friendly delivery methods had the highest levels of bias and misinformation with an average GQS score of 2.36. This is because these videos sacrificed medical accuracy for high comprehensibility. Animations represented 50% (9/18) of this category and were often rife with misleading descriptions of the CABG procedure. This was not surprising as previous studies have revealed the low educational quality of medical animations [19,30]. Similarly, patient accounts were highly biased, which is concerning due to their strong influence on patient decision-making [31]. Furthermore, overly simplistic medical content preferentially attracts viewers with the lowest health literacies, thereby amplifying the spread of misinformation [31,32]. Conversely, live CABG procedures were very medically accurate but lacked any explanation of the video content. Therefore, these videos only received a slightly higher GQS score of 2.95 as they were incomprehensible to individuals without an extensive background in healthcare. Finally, videos formatted as presentations had the best educational quality with an average GQS score of 3.35. This is because these videos found a balance between comprehensibility and medical accuracy and are therefore the best option for patients looking to educate themselves on CABG. However, this content is still well below optimal quality, often omitting outside references and areas of uncertainty. Despite these shortcomings, these results can be used by astute clinicians to effectively guide their patients toward higher-quality videos. In the future, we must incentivize the expansion of the current CABG catalog on YouTube, with a special emphasis on creating videos that do not sacrifice comprehensibility for medical accuracy.

Videos that contained a physician verifying the presented content had a higher average GQS score than those that did not contain a physician. This finding was expected, as it agreed with previous studies on this topic [19,30]. Doctors were more likely to present alternative treatment options, risks, benefits, and accurate information than their non-physician counterparts. However, the average GQS score of videos containing a physician was only 3.16, which is still far from sufficient for proper patient education. This finding reinforces the need for more clinicians to produce higher-quality videos aimed at educating patients on CABG. Such action will enable patients to make cogent medical decisions that improve healthcare outcomes [33].

When videos were stratified by those with lower versus higher engagement and popularity measures (i.e., like-to-dislike ratio and view ratio respectively), no significant differences were found in average GQS scores. This reveals a clear disconnect between community approval and the educational quality of YouTube CABG videos. Numerous previous studies with conflicting evidence regarding engagement parameters and YouTube content quality are present in the literature [7,8,25,31,34-36]. Considering these inconsistent findings, video metrics such as like ratios and view ratios are not reliable guides when judging the educational quality of YouTube healthcare content. This is alarming as YouTube’s algorithm heavily weighs these engagement parameters when calculating which video to suggest to registered users [36,37]. Therefore, it is necessary for physicians to actively direct patients toward videos of higher educational quality instead of relying on video metrics.

Analysis of video duration yielded no significant correlation with average GQS scores, as seen in previous studies [38,39]. However, some authors have revealed positive correlations between video length and educational quality [19,34]. A plausible explanation for these conflicting results is the differences in video content among the aforementioned studies. Some healthcare topics may require detailed explanations that are not sufficiently covered in shorter videos. Other topics, such as CABG content, may be low in educational quality irrespective of the video duration. Days since upload were also not significantly correlated with average GQS scores. Therefore, the quality of CABG videos on YouTube has not meaningfully improved over time. This highlights the need for clinicians and reputable institutions to begin uploading higher-quality videos and rectify this trend.

Despite the current paucity of high-quality CABG content on YouTube, the platform has potential as a reputable educational source. Research has shown that increased patient volumes, physician shortages, and fee-for-service payment models have decreased doctor visit durations; leaving many patients inadequately educated on important healthcare issues [40-42]. YouTube recently has taken steps to reduce the spread of misinformation on the site. On January 26, 2021, YouTube removed over half a million videos containing misleading information about COVID-19 [43]. Furthermore, the platform has partnered with Dr. Garth Graham and numerous credible healthcare institutions to create high-quality medical content [44]. Future studies with longitudinal analysis must be done to evaluate the effect these policy changes have on the quality of health information on YouTube. Other popular social media platforms such as Meta (Meta Platforms, Inc., Menlo Park, California, United States) and TikTok (ByteDance Ltd., Beijing, China) should also be considered in follow-up studies on this topic. Furthermore, despite this effort, medical professionals will still need to play a large role in ensuring proper patient education. Being a profit-driven enterprise, YouTube is susceptible to influences from shareholders who prioritize financial gains. Consequently, it is vulnerable to biases, and depending solely on content moderation may not be a reliable method to achieve an online environment free of misinformation. Ultimately, physicians are the ones who establish trust and relationships with their patients and therefore have the responsibility to guide them towards reputable sources of information.

Limitations

Regarding the limitations of this study, YouTube is a dynamic platform that is growing every day. Thus, video metrics taken at one point in time will not accurately represent those video parameters at a later point. This limitation would be present in any study of a similar design and was minimized by having both independent reviewers record video metrics on the same day. Furthermore, videos below 25,000 views were not evaluated as these were less likely to be watched by patients; however, this may have excluded popular content that was uploaded recently. Another limitation was that videos included in the study were only in English which may reduce its generalizability to other countries. However, English is considered the universal language across the world [22]. Additionally, YouTube was the only video platform analyzed, while other social media sites were not evaluated. This may have excluded videos of higher quality that were present on medical education websites as well. Confirmation bias must also be kept in mind when evaluating the scoring methodology used. Given that videos containing a physician are innately perceived to be more reliable, the GQS score may have subtly been affected by this preconception. Furthermore, the legitimacy of the physicians present in the videos was not scrutinized during this study and is an additional limiting factor to consider when repeating this analysis in the future. One other inherent constraint of this study lies in the profit-centric framework of YouTube as a platform. YouTube primarily generates revenue through video advertisements, making it susceptible to pressures from investors who emphasize profit [45]. Each instance of an ad being viewed or clicked results in charges to advertisers, ranging from $0.10 to $0.30 [46]. Consequently, YouTube’s business model might render its content susceptible to biases, favoring video watchability over educational quality. Finally, the ICC only found moderate agreeability between the independent reviewers. This reduces the reproducibility of this study. Despite these limitations, this study successfully revealed that YouTube is an inadequate educational source for patients learning about the CABG procedure.

## Conclusions

Although CABG videos are readily available on YouTube, this study revealed that they contain considerable biases and misleading information. This is troublesome as patients are increasingly utilizing the internet to make informed medical decisions. However, clinicians who understand the educational landscape of CABG content on YouTube can effectively guide their patients toward reliable videos. Important interventions such as increased physician authorship of videos and more guidance for patients in the online space could potentially pave the way for online videos to effectively offer appropriate patient education in the future.
